# A review of current knowledge of myeloproliferative disorders in the horse

**DOI:** 10.1186/s13028-021-00573-3

**Published:** 2021-02-23

**Authors:** Katy Satué, Juan Carlos Gardon, Ana Muñoz

**Affiliations:** 1 Department of Animal Medicine and Surgery, Faculty of Veterinary, Cardenal Herrera University, Tirant Lo Blanc, 7, Alfara del Patriarca, 46115 Valencia, Spain; 2 Department of Animal Medicine and Surgery, School of Veterinary and Experimental Sciences, Catholic University of Valencia-San Vicente Mártir, Guillem de Castro, 106, 46003 Valencia, Spain; 3 Department of Animal Medicine and Surgery, Faculty of Veterinary, University of Córdoba, University Campus of Rabanales, Ctra. Madrid-Cádiz, 14014 Córdoba, Spain

**Keywords:** Horse, Leukemia, Myeloproliferative neoplasms

## Abstract

Myeloid disorders are conditions being characterized by abnormal proliferation and development of myeloid lineage including granulocytes (neutrophils, eosinophils and basophils), monocytes, erythroids, and megakaryocytes precursor cells. Myeloid leukemia, based on clinical presentation and proliferative rate of neoplastic cells, is divided into acute (AML) and myeloproliferative neoplasms (MPN). The most commonly myeloid leukemia reported in horses are AML-M4 (myelomonocytic) and AML-M5 (monocytic). Isolated cases of AML-M6B (acute erythroid leukemia), and chronic granulocytic leukemia have also been reported. Additionally, bone marrow disorders with dysplastic alterations and ineffective hematopoiesis affecting single or multiple cell lineages or myelodysplastic diseases (MDS), have also been reported in horses. MDSs have increased myeloblasts numbers in blood or bone marrow, although less than 20%, which is the minimum level required for diagnosis of AML. This review performed a detailed description of the current state of knowlegde of the myeloproliferative disorders in horses following the criteria established by the World Health Organization.

## Background

Leukemia is defined as a progressive malignant disease of blood-forming organs, characterized by abnormal proliferation and development of leukocytes and their precursors in blood and bone marrow. Depending on the proportion of blast cells, cell morphology, and expression of specific antigens in the neoplastic cells, leukemia is divided into lymphocytic and myeloid types, and both of them could be acute and chronic [1–4]. Myeloid leukemia is further categorized into acute myeloid leukemia (AML), myeloproliferative neoplasms (MPN, formerly called chronic myeloid leukemia), myelodysplastic syndrome (MDS), and MDS/MPN based on specific clinicopathologic criteria in humans [5]. Since similar entities are also recognized in animals, this classification scheme has been adapted for veterinary medicine [6].

AML is a clonal expansion of myeloid stem cells, which either minimally, partially, or fully differentiation into a particular myeloid lineage (erythroid, megakaryocytic, granulocytic, monocytic). Often, AML is an aggressive rapidly progressive condition with excessive numbers of neoplastic undifferentiated or blast cells which exceed 20% (formerly 30%) of bone marrow or blood cells. These immature cells are increased in bone marrow and in some cases, they might circulate in blood. Aspirate biopsies or necropsy studies of bone marrow can provide cytologic details of the cell population, which might help to identify cell lineage and dysplastic changes [4].

Myeloproliferative neoplasms (MPN, formerly chronic myeloproliferative leukemia CML) occurs when the neoplastic transformation occurs at a later stage of differentiation, when cells are mature. In MPN, the cells resemble the mature cells of the affected lineage, but they appear increased in numbers in blood or/and bone marrow. Typically, MPN is less aggressive than AML, with slower progression, since that the neoplastic transformation occurs at a later stage of differentiation. However, the division between AML and MPN is not absolute. Some cases share characteristics of both and also some chronic type leukemias could progress to acute disease or "blast cell crisis [7, 8]. In addition, based on the presence or absence of abnormal cells in peripheral blood, leukemic, subleukemic and aleukemic leukemia are described. In the leukemic leukemia, abnormal blood cell numbers are increased in blood; in subleukemic leukemia there are increased numbers of blast cells in blood, but the total number of white blood cells is within the reference range and in the aleukemic leukemia, there are no abnormal cells in peripheral blood although abnormal bone marrow findings can be found [9]. Blast cells are uncommonly found in blood in horses [4].

MDS is a disorder characterized by dysplastic changes (i.e., morphologic abnormalities) in one or more cell lineages (erythroid, myeloid and/or megakaryocytic) with a hypercellular marrow, blasts count lower than 20% and concurrent peripheral cytopenias due to ineffective hematopoiesis. Some authors consider MDS as a predecessor for the development of leukemia [10–12].

Identification of different subtypes of AML, MPN, and MDS cases can be performed by cytopathology, blood, and bone marrow cells morphologic findings, cytochemical staining used to identify myeloid cell lineage, histopathology, immunologic expression of cell antigens identified with immunoassays such as immunohistochemistry and flow cytometry [4]. These last techniques are based on the expression of cellular antigens. GM-CSF or IL-4 are essential cytokines to identify myeloid cells. Surface markers such as CD1, CD14, CD69, CD163, and CD206 allow the differentiation between monocytes and macrophages [13]. Other surface antigens, such as EqCD13 allow the identification of myeloid precursors [14]. Table 1 shows the main cytochemical stains used to identify the myeloid cell lineage.

**Table 1:** Cytochemical stains used to identify the lineage of myeloid cells [6, 46]

Cytochemical reaction stain	Cellular element stained	Blasts/cell type identified
Myeloperoxidase (MPO)	Neutrophil primary granules	Myeloblasts strong + /monoblasts faint + Neutrophil; Monocyte
Sudan Blanck B (SBB)	Phospholipids	Myeloblasts strong + /monoblasts faint + Neutrophil; Monocyte
Naphtol AS-D chloroacetate esterase (CAE); specific	Esterese enzymes	Myeloblasts strong + Granulocytes
Leukocyte alkaline phosphatase (ALP)	Neutrophil secondary granules	Metamyelocytes, band and segmented neutrophil Monocyte
Alpha n*aphthyl* acetate esterase (ANAE); non-specific Alpha n*aphthyl* butyrate esterase (ANBE); non-specific	Esterase enzymes	Monoblasts strong + Monocyte strong +

This review reviews the current state-of-art of clinical cases of myelopoietic neoplasias in the horse, classified following the guidelines established by the World Health Organization (WHO) for myeloid leukemias.

### Search strategy

The search for information was initially carried out in the PubMed database (http://www.ncbi.nlm.nih.gov/pubmed) using the words "myeloid leukemia, myeloproliferative neoplasm, myeloproliferative neoplasia, equine, horse". The titles and abstracts were evaluated, expanding in detail the articles referring to these disorders. As additional sources, own studies were used. The experience of the authors in this area has made it possible to adapt the cases described in the literature to the current categorization criteria.

## Review

In this review of equine myeloproliferative disorders, the WHO classification giudelines for for myeloid leukemias [5] are applied. The disorders are therefore divided into acute myeloid leukemias, myeloproliferative neoplasms, and myelodysplastic syndromes with a further subgrouping when applicable.

### Acute myeloid leukemia

In the early 1990s, the Animal Leukemia Study Group (ALSG) described a classification of myeloid leukemia in veterinary medicine. This classification adopted the criteria and definitions of the French-American-British Cooperative Group (FAB) and the National Cancer Institute workshop used for human medicine.

The common terminology for AML classification includes 8 categories (M0 to M8) and was based on the morphological and cytochemical characteristics of blast cells. Lately, cytochemical staining was replaced by immunophenotypic characterization of the cell lineage. Subsequently, the WHO revised this classification and added two new criteria such as chromosomal translocations and evidence of dysplasia [15]. AML is usually diagnosed from a bone marrow aspirate when more than 30% of the nucleated cells are blast cells and more than 50% are non-erythroid cells [3]. However, WHO reviews reduced the invasion of blast cells threshold from 30 to 20% to diagnose, expand, and redefine the different categories of AML [5, 15].

### Acute myeloid leukemia

AML-M0 (acute myeloid leukemia) is diagnosed when blast cell number is greater than or equal to 90% of myeloid cells. Less than or equal to 5% of blast cells in general circulation are stained with myeloperoxidase (MPO) and no Auer blast cells are observed. Auer blast cells are groups of azurophilic granular material forming elongated needles (Auer rods) in the cytoplasm of myeloid leukemic blast cells. To the authors’ knowledge, this subtype has not been documented in the horse yet [15]. AML-M0 diagnosis requires ultrastructural detection of lysosomes containing peroxidase, or immunophenotyping for diagnosis.

A separate entity in the context of AML es the acute undifferentiated leukemia (AUL). Based on FAB and WHO, AUL is characterized by the presence of blastic hematopoietic cell within the bone marrow and/or blood at proportions greater than 20–30%, even up to 100% of the total nucleated cells. Because of the immaturity and atypia of these blasts, differentiation of the cells on morphological, cytochemical and phenotypic characteristics into lymphoid or myeloid is difficult. In human medicine, AUL which is positive for myeloid markers is classified as AML-M0, whereas cases negative for lymphoid and myeloid markers are classified as ‘undifferentiated’ or better still, ‘unclassifiable’ leukemia. In veterinary medicine, AUL is extremely uncommon, although it has been reported in dogs [16].

### Acute myeloblastic leukemia

Acute myeloblastic leukemia (AML-M1 and M2) may be granulocytic or neutrophil and is subdivided into M1 where > 90% of non-erythroid cells are myeloblasts, and M2 when 30–90% of non-erythroid cells are myeloblasts [3]. AML-M1 is the acute myeloproliferative leukemia without maturation. Blast cells are predominant in circulating blood and bone marrow with less than 10% cytoplasmic granulation; at least 5% of the population of malignant blast cells stained with Sudan Black B (SBB) and MPO and, Auer rods may be present. AML-M2 is the AML with maturation. Approximately 30–90% of myeloid cells are blast cells, with at least 10% of neoplastic cells showing maturation (promyelocytes or more); at least 50% stained with MPO and may have Auer rods. In AML-M2, chloroacetate esterase (CAE), α-naphthyl butyrate esterase (ANBE), alkaline phosphatase (ALP), and SBB on peripheral blood smears revealed an 94% of blast cells positives for CAE, occasional positive cells for ALP, and blast cells negative for NBE and SBB [15]. AML-M2 was reported in an 18-year-old Morgan mare after 2 weeks of weight loss, loss of appetite, and depression. Hematological analysis showed pancytopenia, with mild normocytic anemia, marked thrombocytopenia and leukopenia due to neutropenia and lymphopenia and hyperfibrinogenemia. The predominant leukocyte type was a blast cell present at 1.6 × 10^9^/L. In bone marrow aspirate, the differential cell count was composed of 42% lymphoid cells, 5% differentiated myeloid cells, 24% erythroid cells (predominantly rubricytes and metarubricytes), and 29% blast cells with occasional plasma cells [17]. Because blast cells were difficult to differentiate based on morphology, cytochemical stains were used to determine the cell lineage to classify this process [18].

### Acute promyelocytic leukemia

In AML-M3 or promyelocytic, there is a predominance of promyelocytes in both circulating blood and bone marrow [15]. AML-M3 in the FAB system is associated with recurrent genetic abnormalities in humans and it has not been reported in animal species.

### Acute myelomonocytic leukemia

Both granulocyte and monocyte differentiation occur in AML-M4 or myelomonocytic. At least 20% of both tumor cell lines stain for neutrophil or the monocyte series; at least 20% of blast cells in blood or bone marrow and at least 20% of bone marrow cells must be of the monocyte lineage to distinguish AML-M2 from AML-M4 [3]. The percentage of neutrophils and monocytes will vary depending on the stage of the disease with the predominance of either cell. There have been reported cases of AML-M4 in horses of different breeds such as Standardbred Trotters, Thoroughbreds, Quarter Horses, and Hessians horses [12, 14, 19–24]. In these animals, various clinical signs have been described including fever, decreased exercise tolerance, infections that do not respond to antimicrobial therapy, depression, edema, petechiae, weight loss, epistaxis, pneumonia, and colic [19–21, 25, 26]. The most common hematological findings in AML-M4 were anemia, thrombocytopenia, and leukocytosis, normal number of leukocytes or leukopenia, with blast cells or monocytoid cells [23]. Bone marrow aspirates show abundant immature myeloid cells and a high ratio of myeloid to erythroid (M:E ratio) [19, 21, 22]. Brumbaugh et al. [22] presented the case of a 5-year-old Quarter Horse with a bone marrow M:E ratio of 30.5:1, absence of megakaryocytes, and severe clotting disorders. Similarly, Bienzle et al. [19] described absolute megakaryocytic hypoplasia, erythroid hypoplasia, granulocyte reserve depletion, a predominance of immature blast cells-like leukocytes, and an M:E ratio of 50:1. Cooper et al. [4] diagnosed 6 cases of AML, based on predominance of blasts, lack of granulocytic or monocytic differentiation and detection of CD3, CD20 and/or CD79a antigens by immunohistochemistry. Six of these changes were classified as AML with myelomonocytic (n = 4), basophilic (n = 1) and eosinophilic (n = 1) differentiation, based on partial leukocytic differentiation. Blood smears showed anemia, thrombocytopenia, neutropenia, and blast cells without leukocytosis. Bone marrow cellularity ranged from 30 to 100%, and the blast cell ratio ranged from 30 to 60% in AML. Cytochemistry is essential to classify monocytic leukemias [22] since the stain positive with α-naphthyl acetate esterase (ANAE) indicates the monocytic origin of the cells. Additionally, electron microscopy might reveal that monocytoid cells resemble peripheral blood monocytes [19].

### Acute monocytic leukemia

AML-M5 or monocytic is characterized by peripheral monocytosis with neoplastic monocytes comprising more than 80% of nonerythroid cells in the bone marrow [3]. Two subtypes of AML M5 have been described, AML-M5A, with a predominance of monoblasts (> 80%) and AML-M5B with a mixture of monoblasts and promonocytes and ≤ 80% blasts [15]. Most horses reported with AML have had either AML-M4 or AML-M5 [11]. In the horse, several cases of AML-M5 have been reported. Normochromic normocytic anemia, thrombocytopenia, and leukocytosis, characterized by a population of atypical mononuclear cells with monocytoid appearance, absolute neutropenia, and normal numbers of lymphocyte, have been described in a Hessian grey gelding mare [27] and in an Appaloosa mare [28]. Both animals showed a lack of all normal hematopoietic precursors in bone marrow. There were also rare megakaryocytes and very few granulocyte series’ cells. The cells that most closely resembled rubricytes accounted for 45% of the cell population. Most of the cells were large with high nuclear:cytoplasmic ratio and a finely granular basophilic cytoplasm. Some of these cells had clear perinuclear areas that were weak. These cells were morphologically similar to those seen in peripheral blood. Cytochemical staining revealed that most, over 80%, were diffusely positive for ANAE, 10% to 15% were slightly positive for SBB and ALP, which was consistent with the diagnosis of AML-M5 (Table 1). Mitotic figures were present in the atypical cell populations found in lymph node and bone marrow. In addition, Latimer and White [29] identified AML-M5A in a Standardbred 17-year-old gelding with signs of lethargy, intermittent fever, limb edema, increased lung sounds, and submandibular lymphadenopathy. Hematological findings included moderately severe anemia, thrombocytopenia, white blood cell count within the reference range, but with neutropenia and numerous blast cells. Using Wright-Leishman staining and bone marrow smear, a monocyte lineage of the cell population was recognized. In ultra-structural buffy coat preparations, neoplastic monoblasts had one to two nuclei, scattered chromatin, elongated mitochondria, scattered profiles of rough endoplasmic reticulum, microfilament bundles, and pseudopods. The most differentiated monocytoid cells had rare lysosomal granules.

### Acute erythroid leukemia

AML-M6 or acute erythroid leukemia includes two subtypes of erythroid/myeloid leukemia (AML-M6A) and pure erythroid leukemia (AML-M6B). In AML-M6A there is a dual lineage with a co-production of myeloblasts and erythroblasts. More than 50% of the total nucleated cells in the bone marrow are erythroids and at least 30% are myeloblast precursors [3]. Erythroid leukemia or AML-M6B represents a rare form of leukemia in horses and is occasionally reported in dogs [30]. Forbes et al. [31] identified an AML-M6B in a 10-week-old foal, referred for long-duration anemia. Hematology revealed severe anemia and panleukopenia. Cytological examination of bone marrow smears showed an M:E < 0.02:1 (range 0.5–2.4:1.0) ratio with an abundance of red blood cell precursors. The erythroid cell population included rubric, prorubricants, and rubricants, with only a small number of metarubricants present. It was also found numerous mitotic erythroid cells, some of which were atypical and megaloblastic. Later, Panziera et al. [32] described a case of AML-M6B in a 1-year-old filly. Clinically, the animal showed progressive weight loss, markedly pale mucous membranes, and exercise intolerance. Hematological findings were severe anemia, neutropenia, and leukopenia. Cytological evaluation of bone marrow revealed inversion of the M:E ratio (0.2), with 48% of the nucleated cells corresponding to blasts, eccentric nuclei with loose chromatin and inconspicuous nucleoli (1–4 per cell), and abundant and intensely basophilic cytoplasm. These cells were identified as rubriblasts. In some blasts a small clear area adjacent to the nuclei was observed. The percentage of rubricytes and metarubricytes was lower than expected, 8 and 14% respectively. The authors did not observe dysplastic features and mitotic figures were frequently found. In addition to the raw evidence of anemia, the necropsy findings consisted of splenomegaly and lymphadenomegaly, with presence of the same cell populations that those found in bone marrow.

### Acute megakaryocytic leukemia

AML-M7 or megakaryocytic is diagnosed when at least 30% of the nucleated cell population within the bone marrow are megakaryocytes [3]. Megakaryocytic leukemia has been described in dogs [33], but not in cats or horses.

### Acute basophilic leukemia

Acute basophilic leukemia (ABL; M8), although it a rare form of AML, has been reported in dogs [34], cats [35], in a calf [36], and in a horse [37]. Furness et al. [37] described an AML with basophilic differentiation in a 3-year-old gelding Standardbred horse with a history of fever, persistent oral bleeding, and inflammation at the junction of the caudal aspect of the mandibular rami and the proximal neck. Blood analysis showed anemia, leukopenia and severe neutropenia. Bone marrow aspiration was poorly cellular, and the predominant cell was a population of atypical round cells containing few to many basophilic granules. These cells were large with pale basophilic cytoplasm and a moderate nuclear:cytoplasmic ratio. Differentiation was limited, as occasional cells with indented or segmented nuclei contained similar granules. Other cell lines were minimally represented, although a few erythroid precursors were observed. Staining additional aspirates with toluidine blue confirmed metachromatic granules typical of mast cells or basophils, as the positive blue granules are not present in cells of lymphocytic or monocytic origin. Myeloid lineage cells with basophilic differentiation were therefore, suspected.

### Myeloproliferative neoplasms

Myeloproliferative neoplasms or CML have primarily mature cells in blood and/or bone marrow, without a prominent increase in blast cells. CML is commonly found in adult animals. Although these proceses are chronic in nature, they can progress to AML. Subgroups of this neoplasm are quite rare in horses and include [3].

#### Chronic granulocytic leukemia

Chronic granulocytic leukemia (CGL) is characterized by a proliferation of cell lineages of relatively mature neutrophils, eosinophils, or basophils. Within the bone marrow, relatively mature granulocytes predominate, with an M:E ratio from 5:1 to 20:1 [3]. An early report of CGL was published in a 9-year-old Quarter Horse with progressive weight loss, pneumonia, and loss of appetite over 47 days. The most remarkable abnormality in blood was a persistent neutropenia and circulating myelocytes and metamyelocytes. Many of these cells appeared to have mature neutrophil cytoplasm, but they had round nuclei. In addition, the authors described large fragments of granulocytic cell cytoplasm in blood. In bone marrow, the authors described absence of granulocyte post-mitotic maturation, with a predominance of cells with a medium to large round central or eccentric nuclei and pale cytoplasm, that stained positively for peroxidase. Because of the time this study was reported, additional diagnostic methods were not performed [38].

More recently, Johansson et al. [39] reported a case of CGL in a 4-year-old Swedish Warmblood. The blood smear of this animal revealed normochromic, microcytic anemia with anisocytosis, leukocytosis with neutrophilia, left shift, toxic changes in neutrophils, monocytosis, and thrombocytosis. In addition, abnormal circulating red blood cells, mild poikilocytosis, marked anisocytosis, and nucleated red blood cells were found. Bone marrow aspiration from the sternum showed a slight increase in M:E ratio (4.0, 0.5–3.76). Although the morphology and maturation gradient of myeloid and erythroid cells were mostly inconspicuous, some immature and dysplastic myeloid cells were observed. Erythroid hypoplasia and megakaryocyte hypoplasia were consistent with anemia and thrombocytopenia. In these horses, bleeding secondary to coagulopathy or to immune-mediated mechanisms was a common finding. Biopsies of the submandibular lymph nodes revealed dizzy infiltration of myeloid cells destroying normal architectural detail. Unlike abscessed lymph nodes, in CGL, myeloid cells are not primarily mature neutrophils.

Neutrophilic leukemia, a subtype of MPN, has been described in horses with progressive loss of physical condition, anorexia, limb edema, fever, clotting disorders, icterus from hemolytic anemia, and recurrent infections [9, 40]. Characteristic laboratory findings of neutrophilic leukemia in horses include progressive normochromic normocytic anemia [9, 38] and marked thrombocytopenia [9]. These characteristics were consistent with an impaired production in the bone marrow due to marked myelogenous proliferation.

#### Chronic eosinophilic leukemia

Chronic eosinophilic leukemia (CEL) is a clonal proliferation of eosinophilic precursors that might result in a persistently high number of eosinophils in blood, bone marrow, or tissues [41]. Although some authors consider CEL a subtype of a myeloproliferative variant of hypereosinophilic syndrome, others consider it an independent entity [15]. In a 10-month-old Standardbred colt with edema and hemorrhagic diathesis, CEL was identified. The foal had severe anemia, thrombocytopenia, mild hypoproteinemia, and marked eosinophilia, with circulating immature or atypical eosinophils. Bone marrow aspiration showed atypical eosinophilic precursors, with few erythroid precursors and absence of megakaryocytes [42]. Postmortem histological examination of the animal revealed infiltrations of atypical eosinophils and eosinophilopoiesis in the spleen. The disease was similar to the idiopathic hypereosinophilic syndrome in horses [43], but there was no cardiac and neurological compromise typical of hypereosinophilic syndrome.

#### Chronic basophilic leukemia

Chronic basophilic leukemia (CBL) is an extremely rare disease, described in humans [44], in one cat [45] and in a dog [46], but to the authors’ best knowledge, it has not been reported in horses. In the cat, leukocytosis, mature neutrophilia, eosinophilia, and basophilia were observed. The bone marrow had hypercellular particles with an adequate number of megakaryocytes and 43% of the total nucleated population were mature basophils. Blast cells accounted for 17% of all nucleated cells. Cytochemical staining with omega-exonuclease, a specific marker for basophils, and CAE and SBB indicate basophilic granulocyte origin. Based on these findings, CBL was diagnosed [45]. In the dog, bone marrow revealed numerous basophilic cells with a round or segmented nucleus and cytoplasm with basophilic granules exhibiting metachromasia on toluidine blue staining [46].

#### Polycythemia vera or primary erythrocytosis

Polycythemia vera or primary erythrocytosis is rare in horses. It is the result of the malignant transformation of bone marrow precursors of all bone marrow cell lines and manifests itself with erythrocytosis. It may be accompanied by thrombocytosis and leukocytosis, but serum erythropoietin (EPO) is typically normal [47]. In horses, neoplastic conditions in which red blood cell production is autonomous and independent of EPO concentrations are limited. McFarlane et al. [48] reported a primary erythrocytosis in a 2-year-old Arabian gelding. Cytologic evaluation of the bone marrow revealed a predominance of erythroid precursors in an orderly maturation sequence. The authors diagnosed a primary erythrocytosis based on normal serum EPO concentrations, absence of neoplasia and mild hipoxemia. Additional diagnostic procedures were not performed in this case. Later, Steiger and Feiger [49] identified a supposed polycythemia vera in a 13-year-old Thoroughbred gelding. This horse had concomitantly, granular cell pulmonary myoblastoma, severe liver fibrosis, mild acute tubular nephrosis, and thrombosis of the abdominal aorta. The possibility of secondary polycythtemia due to the lung neoplasia was not entirely excluded, but the authors considered this possibility unlikely.

In humans, polycythemia vera is due to the mutation (V1617F) in the pseudokinase domain of Janus-activated kinase 2 (*JAK2*). *JAK2* is a nonreceptor tyrosine kinase that downstream of the EPO receptor that directs the signaling cascade triggered by the binding of EPO to its receptor in red blood cell precursors in the bone marrow. In this way, signaling promotes cell division and erythropoiesis. Normally, *JAK2* is only activated, by phosphorylation of tyrosine residues, when the EPO binds to its receptor. However, up to 90% of patients with polycythemia vera have a homozygous mutation of a single amino acid in *JAK2*. In these cases, the valine is replaced by phenylalanine in the amino acid 617, which makes the enzyme constitutively active. This leads to erythropoiesis, either independently of EPO or because the precursors become hypersensitive to normal EPO concentrations [50]. Polycythemia vera is rarely reported in animals, even though, a genetic mutation in *JAK2* has been identified in dogs [51].

#### Essential thrombocythemia

Essential thrombocythemia (ET) represents an overproduction of megakaryocytes in bone marrow that might result in the release of abundant platelets into blood. These high amounts of platelets may not be completely functional, and might cause vascular thrombosis and bleeding disorders. In humans with ET, a mutation of the *JAK2* gene in blood cells has been described [52]. In cats and dogs, ET has been described [53–55]. In these animals, exercise tolerance was reduced, and pale mucous membranes were present without blood loss. They also had moderate to severe anemia and marked thrombocytosis (> 1.249 × 10^9^/L) with a positive Coombs test. Peripheral blood smear revealed the presence of basophilia and a large number of abnormally shaped megakaryocytes was found in bone marrow. The diagnosis of ET should only be considered in animals with sustained and unexplained thrombocytosis longer than 1–2 months and without evidence of MPN (low myeloblasts, and without dysplasia, leukocytosis, or severe anemia). The presence of sensitive markers of inflammation (e.g. acute reactive proteins) might help to distinguish between reactive thrombocytosis and ET [54, 55].

#### Chronic monocytic leukemia

Chronic monocytic leukemia (CMoL) is a clonal stem cell disorder characterized by an excessive proliferation of granulocytic and monocytic cells. A case of CMoL was reported in a 6-year-old Hessian gray gelding with a history of impaired performance, cough, colic, edema of the ventral abdomen, prepuce and limbs, reduced skin turgor, pale mucous membranes, forced costoabdominal breathing, reduced venous return, enlarged lymph nodes, and splenomegaly. The hematological analysis revealed anemia, leukocytosis, and a high percentage of monocytoid leukemic cells. Generalized lymphadenopathy, splenomegaly, ascites, hydrothorax, and a diffusely thickened gut wall were found at necropsy. Massive infiltration with monocytoid leukemic cells was detected in lymph nodes, spleen, bone marrow, liver, gut wall, kidneys, and choroid plexus. Incubation of living cells obtained from a leukocyte concentrate with latex particles revealed phagocytosis in the leukemic cells on light and electron microscopy. The leukemic cells were marked mainly with ANBE and CAE, showing their monocytic origin (Table 1). On scanning electron microscopy, the leukemic cells had prominent ruffles and ridge-like profiles compatible with CMoL [26].

#### Chronic myelomonocytic leukemia

Chronic myelomonocytic leukemia (CMMoL) is often included among MDS because of its multilinear dyshematopoiesis and its tendency to progress to AML. In cats and dogs, CMMoL usually has extreme leukocytosis (> 1 × 10^9^/L), with neutrophilia and monocytosis and a pronounced leftward shift in both cell series. Circulating myeloid blast cells can represent between 5 and 10% of leukocytes. Hypersegmented granulocytes and monocytes are frequently found, as well as mild to severe normochromic macrocytic anemia and thrombocytopenia. As with CMoL, repeated blood and bone marrow tests with special staining or immunophenotyping are needed to confirm the diagnosis of CMMoL [56]. In dogs and cats, CMMoL appear to be more common than CMoL than of CMoL [57–60]. To the authors’ best knowledge, CMMoL has not been described in horses.

Cooper et al. [4] showed membranous expression of Iba-1/IAF-1 (ionized calcium-binding adapter molecule-1/allograft inflammatory factor-1), granulocytic cells (ranging from myelocytes to segmented neutrophils) expressed CD172a. Antibody to Iba-1/IAF-1 appears to label myelomonocytic precursor cells and macrophages. However, in some cases, cells with round nuclei and lacking cytoplasmic vacuoles or granules were also labeled by Iba-1/IAF-1 antibody. This situation suggests that these might be myeloblasts. The bone marrow has an increased proportion of blast cells with partial neutrophilic differentiation, scattered early-stage rubricytes, and near-complete paucity of megakaryocytes. Some myelocytes and most differentiated granulocytes expresses CD172a. Antibody to CD172a, signal regulatory protein-a, labeled some macrophages and all maturing neutrophils from myelocytes to segmented neutrophils in the bone marrow. This antibody did not label eosinophilic or basophilic precursor cells. In equine tissues antibody to CD163 also marks cells with macrophage morphology.

## Myelodysplasic syndromes

The myelodysplasic syndrome (MDS) represents a group of disorders characterized by ineffective hematopoiesis and dysplastic alterations in single to multiple cell lineages with a hypercellular marrow, blasts counts lower than 20% and concurrent peripheral cytopenias due to ineffective hematopoiesis [10]. However, since domestic animals with MDS may develop AML, it has been considered a preleukemic disorder. It has been described in a gelding Quarter Horse [10]. Further, Miglio et al. [12] reported a case of acute myelomonocytic leukemia with myelodysplasia‐related features in a 6-year-old Italian Saddle mare. The combined results of abnormal morphology of circulating neoplastic cells and bone marrow cytology, flow cytometry and cytochemistry analysis allowed the diagnosis of this disorder.

Table 2 shows the classification of myeloproliferative alterations, their main diagnostic characteristics and the cases described in horses.

**Table 2:** Classification of myelocytic leukemia, main characteristics and cases reported in horses

Type of myeloid leukemia		Main characteristics and identification	Equine cases reported in literature
AUL	Acute undifferentiated leukemia	Considered a separate entity of AML-M0. Even up to 100% of the total nucleated cells can be blasts in bone marrow or blood. Highly immature atypic cells. In humans, some AUL are classified as ‘unclassifiable’ leukemia	Not reported
Acute myeloid leukemias			
AML-M0	Acute myeloid leukemia	Blast cells ≥ 90% of myeloid cells. < 5% of blast cells in general circulation are stained with myeloperoxidase. No Auer blast cells. Ultrastructural detection of lysosomes with myeloperoxidase or immunophenotyping required for characterization	Not reported
AML-M1	Acute myeloblastic leukemia (without maturation)	> 90% of the non-erythroid cells are myeloblasts. Blasts with less than 10% cytoplasmic granulation. At least 5% of the population of blasts stained with Sudan Black B and myeloperoxidase. Auer rods may be present	Not reported
AML-M2	Acute myeloblastic leukemia (with maturation)	30–90% of the non-erythroid cells are myeloblasts. At least 10% of the neoplastic cells shows maturation. At least 50% stained with myeloperoxidase and may have Auer rods	Clark et al. [17]
AML-M3	Promyelocytic	Predominance of promyelocytes in blood and bone marrow. Associated with recurrent genetic abnormalities in humans	Not reported
AML-M4	Myelomonocytic	Both granulocyte and monocyte differentiation. > 20% blast cells in blood or bone marrow. > 20% of bone marrow cells must be of monocyte lineage	Bienzle et al. [19] Blue et al. [20] Boudreaux et al. [21] Brumbaugh et al. [22] Buechner-Maxwell et al. [23] Mori et al. [24] Ringger et al. [25] Spiers et al. [26] Miglio et al. [12]
AML-M5	Monocytic	Peripheral monocytosis. Neoplastic monocytes comprise more than 80% of non-erythroid cells in bone marrow. Two subtypes: AML-5A: predominantly monoblasts (> 80%) AML-5B: mixture of monoblasts and promonocytes (< 80% blasts)	Burhardt et al. [27] Monteith and Cole [28] Latimer and White [29]
AML-M6	Acute erythroid leukemia Subtype M6A	Dual lineage with a co-production of myeloblasts and erythroblasts. > 50% of the total nucleated cells in bone marrow are of erythroid lineage; at least 30% myeloblast precursors	Not reported
AML-M6	Acute erythroid leukemia Subtype M6B	Undifferentiated or pronormoblastic immature cells the erythroid lineage > 80%. M: E ratio < 0.02:1	Forbes et al. [32] Panziera et al. [32]
AML M7	Megakaryocytic	Blast cells > 20% of the circulating cells or bone marrow and at least 30% of the marrow cells is of megakaryocyte lineage	Not reported
AML-M8	Acute basophilic leukemia	Stain with toluidine blue. Metachromatic granules typical of mast cells or basophils	Furness et al. [37]
Myeloproliferative neoplasms			
CGL	Chronic granulocytic leukemia	Proliferation of cell lineages of relatively mature neutrophils, eosinophils or basophils Relatively mature granulocytes predominate in blood marrow	Searcy and Orr [38] Johansson et al. [39]
CEL	Chronic eosinophilic leukemia	Proliferation of eosinophil precursors with high number of eosinophils in blood, bone marrow or peripheral tissues. Immature or atypical eosinophils	Morris et al. [42]
CBL	Chronic basophilic leukemia	Increased number of basophils in bone marrow and blood. Blast cells accounted for 17% of all nucleated cells. Positive staining to omega-exonuclease, naphtol AS-D chloroacetate esterase and Sudan Black B	Not reported
Polycythemia vera or primary erythrocytosis		It affects all bone marrow cell lines and manifest itself with erythrocytosis. Sometimes accompanied by thrombocytosis and leukocytosis. Erythropoietin values within normal limits	Steiger and Feiger [49] McFarlane et al. [48]
ET	Essential thrombocythemia	Overproduction of megakaryocytes in bone marrow	Not reported
CMoL	Chronic monocytic leukemia	Excessive proliferation of granulocytic and monocytic cells	Spiers et al. [26]
CMMoL	Chronic myelomonocytic leukemia	Circulating myeloid blast cells can represent 5–10% of the leukocytes. Hypersegmented granulocytes and monocytes	Not reported
Myelodysplasic síndromes			
MDS	Myelodysplastic syndrome	Ineffective hematopoiesis with dysplastic alterations affecting one to multiple cell lineages Hypercellular marrow, blasts count < 20%, concurrent peripheral cytopenia. AML can be developed after MDS	Durando et al. [10] Miglio et al. [12]

## Conclusions

Although uncommon, myeloid leukemias (both acute and chronic) and myelodysplasic syndromes have been reported in horses. The most commonly described myeloid leukemia reported in horses has been AML-M4 (myelomonocytic) and AML-M5 (monocytic). Isolated cases of AML-M5 (monocytic), AML-M6B (acute erythroid leukemia) and chronic granulocytic leukemia have also been reported. Unreported myeloid leukemias are AML-M0 (acute myeloid leukemia), AML-M1 (acute myeloblastic leukemia), AML-M3 (promyelocytic), AML-6A (acute erythroid leukemia), AML-M7 (megakaryocytic), chronic basophilic leukemia, essential thombocythemia and, chronic myelomonocytic leukemia. Until now, the classification of leukemic processes in the horse has combined hematological and bone marrow cytological studies, some stains for the cytochemical study of progenitor and/or mature cells, and scarce markers for the immunophenotypic study of the myeloid series. Unlike what happens in humans and other animal species like dogs and cats, the limited available methodology for the immunohistochemical or cytogenetic study currently makes it difficult to differentiate the different types of myeloid leukemias in the horse. Therefore, future research based on these areas would be needed to correctly classify these malignancies in the equine species.

## Data Availability

The datasets used and/or analysed during the current study are available from the corresponding author on reasonable request.

## Abbreviations

ABL: Acute basophilic leukemia
AML-M6A: Acute erythroid/myeloid leukemia
AML-M7: Acute megakaryocytic leukemia
AML-M5: Acute monocytic leukemia
AML-M1 and M2: Acute myeloblastic leukemia
ALP: Alkaline phosphatase
ALSG: Animal Leukemia Study Group
AML: Acute myeloid leukemia
AML-M1: Acute myeloproliferative leukemia without maturation
AML-M3: Acute promyelocytic leukemia
AML-M4: Acute myelomonocytic leukemia
AML-M6B: Acute pure erythroid leukemia
ANAE: α-Naphthyl acetate esterase
ANBE: α-Naphthyl butyrate esterase
AUL: Acute undifferentiated leukemia
CAE: Chloroacetate esterase
CBL: Chronic basophilic leukemia
CEL: Chronic eosinophilic leukemia
CGL: Chronic granulocytic leukemia
CML: Chronic myeloid leukemia
CMMoL: Chronic myelomonocytic leukemia
CMoL: Chronic monocytic leukemia
EPO: Erythropoietin
ET: Essential thrombocythemia
FAB: French-American-British Cooperative Group
JAK2: Janus-activated kinase 2
M:E ratio: Myeloid to erythroid
MDS: Myelodysplastic disease
MPN: Myeloproliferative neoplasm
MPO: Myeloperoxidase
SBB: Sudan Black B stain
WHO: World Health Organization

## References

[1] Kelton DR, Holbrook TC, Gilliam LL, Rizzi TE, Brosnaham MM, Confer AW (2008). Bone marrow necrosis and myelophthisis: manifestations of T-cell lymphoma in a horse. Vet Clin Pathol..

[2] Muñoz A, Riber C, Trigo P, Castejón F (2009). Hematopoietic neoplasias in horses: myeloproliferative and lymphoproliferative disorders. J Equine Sci.

[3] Bienzle D, Latimer KS (2011). Hematopoietic neoplasia. Duncan and Prasse's Veterinary Laboratory Medicine: Clinical Pathology.

[4] Cooper CJ, Keller SM, Arroyo LG, Hewson J, Kenney D, Bienzle D. Acute leukemia in horses.Vet Pathol*.* 2018;55:159–72. 10.1177/0300985817720983 28812528

[5] Arber DA, Orazi A, Hasserjian R, Thiele J, Borowitz MJ, Le Beau MM (2016). The 2016 revision to the World Health Organization classification of myeloid neoplasms and acute leukemia. Blood.

[6] Valli V, Kiupel M, Bienzle D, Maxie MG (2015). The hematopoietic system. Jubb, Kennedy and Palmer’s Pathology of Domestic Animals.

[7] Jaffe ES, Harris NL, Stein H (2001). World Health Organization classification of tumours: pathology and genetics of tumors of haematopoietic and lymphoid tissues.

[8] Dobson J, Villiers E, Morris J (2006). Diagnosis and management of leukaemia in dogs and cats. Practice.

[9] Savage CJ (1998). Lymphoproliferative and myeloproliferative disorders. Vet Clin North Am Equine Pract..

[10] Durando M, Alleman AR, Harvey JW (1994). Myelodysplastic syndrome in a quarter horse gelding. Equine Vet J.

[11] Taintor J (2012). Equine leukaemia. Equine Vet Educ.

[12] Miglio A, Pepe M, Felippe MJB, Antognoni MT (2019). Subleukaemic acute myeloid leukaemia with myelodysplasia in a horse. Equine Vet Educ.

[13] Steinbach F, Stark R, Ibrahim S, Gawad EA, Ludwig H, Walter J (2005). Molecular cloning and characterization of markers and cytokines for equid myeloid cells. Vet Immunol Immunopathol.

[14] McClure JT, Young KM, Fiste M, Sharkey LC, Lunn DP (2001). Immunophenotypic classification of leukemia in 3 horses. J Vet Intern Med.

[15] McManus PM (2005). Classification of myeloid neoplasms: a comparative review. Vet Clin Pathol.

[16] Miglio A, Antognoni MT, Miniscalco B, Caivano D, Lepri E, Birettoni F, Mangili V (2014). Acute undifferentiated leukaemia in a dog. Aust Vet J.

[17] Clark P, Cornelisse CJ, Schott HC, Swenson CL, Bell TG (1999). Myeloblastic leukaemia in a Morgan horse mare. Equine Vet J.

[18] Raskin RE (2017). Diagnostic tools and dilemmas with equine leukemias. Vet Pathol.

[19] Bienzle D, Hughson SL, Vernau W (1993). Acute myelomonocytic leukemia in a horse. Can Vet J.

[20] Blue J, Perdizet J, Brown E (1987). Pulmonary aspergillosis in a horse with myelomonocytic leukemia. J Am Vet Med Assoc.

[21] Boudreaux MK, Blue IT, Durham SK, Vivrette SL. Intravascular leukostasis in a horse with myelomonocytic leukemia.Vet Pathol.1984;21:544–6. 10.1177/030098588402100521 6592873

[22] Brumbaugh GW, Stitzel KA, Zinkl JG, Feldman BF (1982). Myelomonocytic myeloproliferative disease in a horse. J Am Vet Med Assoc.

[23] Buechner-Maxwell V, Zhang C, Robertson J, Jain NC. Antczak DF, Feldman BF, Murray MJ. Intravascular leukostasis and systemic aspergillosis in a horse with subleukemic acute myelomonocytic leukemia*.* J Vet Intern Med.1994; 8: 258–63. 10.1111/j.1939-1676.1994.tb03229.x 7983620

[24] Mori T, Ishida T, Washizu T, Yamagami IT, Umeda T, Ugiyama MS, Otoyoshi M (1991). Acute Myelomonocytic leukemia in a horse. Vet Pathol.

[25] Ringger NC, Edens L, Bain P, Raskin RE, Larock R (1997). Acute myelogenous leukaemia in a mare. Aust Vet J.

[26] Spiers SJ, Madewell BR, Zinkl JE, Ryan AM (1986). Acute myelomonocytic leukemia in a horse. J Am Vet Med Assoc.

[27] Burkhardt E, von Saldern F, Huskamp B (1984). Monocytic leukemia in a horse. Vet Pathol.

[28] Monteith CN, Cole D (1995). Monocytic leukemia in a horse. Can Vet J.

[29] Latimer KS, White SL (1996). Acute monocytic leukaemia (M5a) in a horse. Comp Haematol Int.

[30] Mylonakis ME, Kritsepi-Konstantinou M, Vernau W, Valli VE, Pardali D, Koutinas AF (2012). Presumptive pure erythroid leukemia in a dog. J Vet Diagn Inv.

[31] Forbes G, Feary DJ, Savage CJ, Nath L, Church S, Lording P (2011). Acute myeloid leukaemia (M6B: pure acute erythroid leukaemia) in a Thoroughbred foal. Aust Vet J.

[32] Panziera W, Tessele B, BianchiI R, Tochetto C, De La Corte F, Brass K, Fighera R (2015). Equine acute erythroid leukemia. Ciência Rural.

[33] Comazzi S, Gelain ME, Bonfanti U, Roccabianca P (2010). Acute megakaryoblastic leukemia in dogs: a report of three cases and review of the literature. J Am Anim Hosp Assoc.

[34] Mears EA, Raskin RE, Legendre AM (1997). Basophilic leukemia in a dog. J Vet Intern Med.

[35] Iwanaga T, Miura N, Miyoshi N, Endo Y, Momoi Y (2012). Abnormal erythroid cell proliferation and myelofibrosis in a cat. J Vet Med Sci.

[36] Takahashi Y, Yoneyama S, Yamamoto S, Shibahara T, Kadota K (2006). Acute basophilic leukaemia in a calf. Vet Rec.

[37] Furness MC, Setlakwe E, Sallaway J, Wood D, Fromstein J, Arroyo LG (2016). Acute myeloid leukemia with basophilic differentiation in a 3-year-old Standardbred gelding. Can Vet J.

[38] Searcy GP, Orr JP (1981). Chronic granulocytic leukemia in a horse. Can Vet J..

[39] Johansson AM, Skidell J, Lilliehöök I, Tvedten HW (2007). Chronic granulocytic leukemia in a horse. J Vet Intern Med.

[40] Latimer KS, Andreasen CB, Cowell R, Tyler R (2002). Bone Marrow. Diagnostic Cytology and Hematology of the Horse.

[41] Kumar A, Sinha S, Tripathi AK (2007). Chronic eosinophilic leukemia: a case report and review of literature. Indian J Hematol Blood Transfus.

[42] Morris DD, Bloom J, Roby KA, Woods K, Tablin F (1984). Eosinophilic myeloproliferative disorder in a horse. J Am Vet Med Assoc.

[43] Schumacher J, Spano JS, Oliver JL, Smith RA (1991). Hypereosinophilic syndrome in an American paint horse. J Equine Vet Sci.

[44] Cehreli C, Ates H, Cehreli R, Sercan Z, Demirkan F (2013). New paraneoplastic syndrome in chronic basophilic leukemia. Int J Hematol.

[45] Mochizuki H, Seki T, Nakahara Y, Tomita A, Takahashi M, Fujino Y (2014). Chronic myelogenous leukaemia with persistent neutrophilia, eosinophilia and basophilia in a cat. J FelineMed Surg.

[46] Azakami A, Saito A, Ochiai K, Ishiwata T, Takahashi K, Kaji N (2019). Chronic basophilic leukaemia in a dog. J Comp Pathol.

[47] Sellon DC, Wise LN, Reed SM, Bayly WM, Sellon DC (2010). Disorders of the hematopoietic system. Equine Internal Medicine.

[48] McFarlane D, Sellon DC, Parker B (1998). Primary erythrocytosis in a 2-year-old Arabian gelding. J Vet Intern Med.

[49] Steiger R, Feige K (1995). Case report: Polycythemia in a horse. Schweiz Arch Tierheilkd.

[50] Spivak JL (2018). Polycythemia vera. Curr Treat Options Oncol.

[51] Beurlet S, Krief P, Sansonetti A, Briend-Marchal A, Kiladjian JJ, Padua RA, Chomienne C, Cassinat B (2011). Identification of JAK2 mutations in canine primary polycythemia. Exp Hematol.

[52] Tefferi A (2011). Annual clinical updates in hematological malignancies: a continuing medical education series: polycythemia vera and essential thrombocythemia: 2011 update on diagnosis, risk-stratification, and management. Am J Hematol.

[53] Chisholm-Chait A (1999). Essential thrombocythemia in dogs and cats Part 1. Comp Cont Educ Vet..

[54] Favier RP, van Leeuwen M, Teske E (1999). Essential thrombocythaemia in two dogs. Comp Cont Educ Pract.

[55] Stokol T, Weiss DJ (2010). Essential thrombocythemia and reactive thrombocytosis. Schalm's Veterinary Hematology.

[56] Hisasue M, Nishimura T, Neo S, Nagashima N, Ishikawa T, Tsuchiya R, Yamada T (2008). A dog with acute myelomonocytic leukemia. J Vet Med Sci.

[57] Hiraoka H, Hisasue M, Nagashima N, Miyama T, Tanimoto T, Watanabe M (2007). A dog with myelodysplastic syndrome: chronic myelomonocytic leukemia. J Vet Med Sci.

[58] Marino CL, Tran JNSN, Stokol T (2017). A typical chronic myeloid leukemia in a German Shepherd dog. J Vet Diagn Invest.

[59] Rossi G, Gelain ME, Foroni S, Comazzi S (2009). Extreme monocytosis in a dog with chronic monocytic leukaemia. Vet Rec.

[60] Steinbach F, Stark R, Ibrahim S, Gawad EA, Ludwig H, Walter J, Commandeur U, Mauel S (2005). Molecular cloning and characterization of markers and cytokines for equid myeloid cells. Vet Immunol Immunopathol.

